# Tau Lysine Pseudomethylation Regulates Microtubule Binding and Enhances Prion-like Tau Aggregation

**DOI:** 10.3390/ijms24098286

**Published:** 2023-05-05

**Authors:** Yuxing Xia, Brach M. Bell, Benoit I. Giasson

**Affiliations:** 1 Department of Neuroscience, College of Medicine, University of Florida, Gainesville, FL 32610, USA; 2 Center for Translational Research in Neurodegenerative Disease, College of Medicine, University of Florida, Gainesville, FL 32610, USA; 3 McKnight Brain Institute, College of Medicine, University of Florida, Gainesville, FL 32610, USA

**Keywords:** Alzheimer’s disease, frontotemporal dementia, tau protein, tauopathies, methylation, aggregation, microtubule binding, post-translational modifications, prion-like spread

## Abstract

Alzheimer’s disease (AD) and frontotemporal dementia (FTD) can be classified as tauopathies, which are a group of neurodegenerative diseases that develop toxic tau aggregates in specific brain regions. These pathological tau inclusions are altered by various post-translational modifications (PTMs) that include phosphorylation, acetylation, and methylation. Tau methylation has emerged as a target of interest for its potential involvement in tau pathomechanisms. Filamentous tau aggregates isolated from patients with AD are methylated at multiple lysine residues, although the exact methyltransferases have not been identified. One strategy to study the site-specific effects of methylation is to create methylation mimetics using a KFC model, which replaces lysine (K) with a hydrophobic group such as phenylalanine (F) to approximate the effects of lysine methylation (C or methyl group). In this study, tau methylmimetics were used to model several functional aspects of tau methylation such as effects on microtubule binding and tau aggregation in cell models. Overall, several tau methylmimetics displayed impaired microtubule binding, and tau methylmimetics enhanced prion-like seeded aggregation in the context of the FTD tau mutation P301L. Like other PTMs, tau methylation is a contributing factor to tau pathogenesis and could be a potential therapeutic drug target for the treatment of different tauopathies.

## 1. Introduction

Alzheimer’s disease (AD) is the most common type of dementia, affecting 6.5 million Americans, and this number is projected to rapidly grow to 13.8 million patients by 2060 [1]. AD is characterized by two major types of protein inclusions in the brain: Aβ amyloid plaques and neurofibrillary tangles comprised of tau protein [2]. Tau protein normally promotes microtubule (MT) assembly and stabilizes MTs [3,4]; however, this can be disrupted in disease states associated with the formation of tau inclusions [2]. Pathological tau aggregates are also present in multiple neurodegenerative disorders collectively known as tauopathies, which include frontotemporal dementia (FTD), progressive supranuclear palsy (PSP), corticobasal degeneration (CBD), and chronic traumatic encephalopathy (CTE) [5,6]. One way to differentiate clinically distinct tauopathies is by mapping the different patterns of protein post-translational modifications (PTM), which can affect both the structural and functional properties of tau protein [7]. Tau is incredibly versatile in a number of PTMs, which include phosphorylation, acetylation, ubiquitination, methylation, and other modifications [7,8,9]. 

Of these PTMs, tau methylation has been less extensively studied, and much is unknown about its effects on tau structures and functions [10]. Very few tau methyltransferases have been identified except for SETD7 lysine methyltransferase [10,11]. Tau methylation patterns are significantly different between cognitively normal and AD brains based on mass spectrometry studies [9,12,13]. Tau fibrils from AD brains also have specific methylation sites that are uniquely elevated, and methylated tau is a component of tau aggregates [13]. However, in vitro studies have shown that bulk reductive methylation can lead to decreased tau aggregation and reduced MT assembly [9]. So far, few studies have examined individual tau methylation sites and their effects on tau functions.

In the current study, the site-specific effects of tau methylation on MT binding and tau aggregation were investigated. One approach to studying PTMs is the use of mimetics to make amino acid changes to approximate the size and charge of the modification of interest, and this has been successful for phosphorylation and acetylation [14,15]. Based on prior studies on the methylation of other proteins [16,17,18], tau methylmimetics were created using a KFC model by mutating lysine (K) into phenylalanine (F), a bulky hydrophobic group, to approximate the major effects of tau lysine methylation (C or methyl group). Overall, the results show that specific tau methylmimetics can directly modulate MT binding and enhance prion-like seeded aggregation of the P301L tau mutant. 

## 2. Results

### 2.1. Site Selection for Methylmimetics

**Figure 1:**
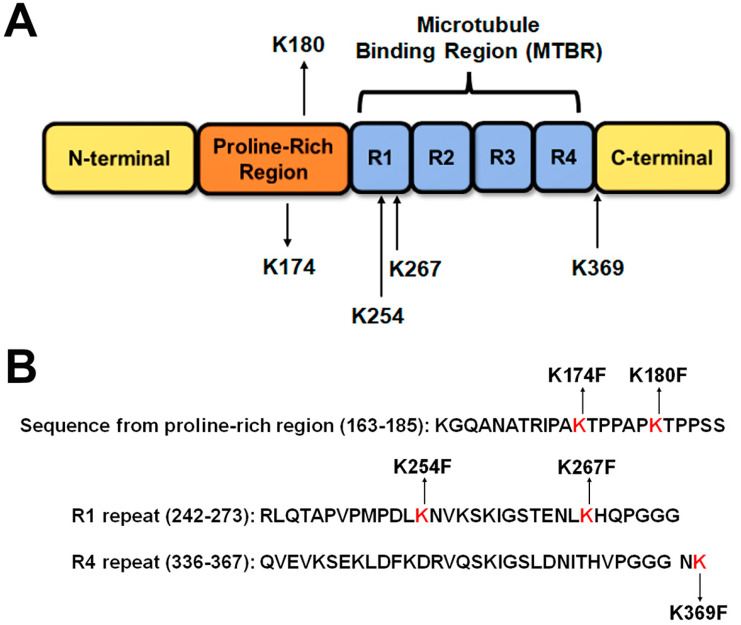
**Overview of major tau methylation sites and methylation mimetics that were used in the study**. (**A**) A diagram of the tau protein sequence depicts different regions of the tau protein, including the N-terminal domain, the proline-rich region, the microtubule-binding region, and the C-terminal domain according to numbering based on the 2N4R human tau isoform. Lysine methylation sites that were examined in this study are shown with their nearby amino acid sequences. R1 to R4 represent the four major MT binding repeats that form the microtubule-binding region of tau. (**B**) Methylmimetics and nearby tau sequences are shown. K174F and K180F are in the proline-rich region. K254F and K267F are in the R1 repeat, while K369F is just outside of the R4 repeat.

A series of tau methylmimetics were selected based on tau lysine methylation sites that were identified from mass spectrometry studies of tau filaments derived from the post-mortem brains of AD patients [13]. These disease-relevant tau methylation sites include K254 and K267, which are the most abundant sites found in tau filaments, but also K174 and K180 [13]. K369 is a potential tau methylation site that is capable of being methylated after in vitro reductive methylation reactions [9]. This site is of particular interest because of its overlap with the K369I tau mutation, which can directly cause familial Pick’s disease [19]. A summary of all the tau methylation sites and the nearby amino acid sequences is shown in more detail in Figure 1.

### 2.2. Tau Methylmimetics Regulate MT Binding

**Figure 2:**
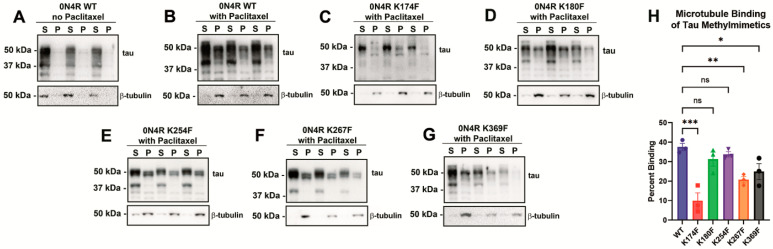
**Tau methylmimetics display site-dependent regulation of MT binding.** A cell-based MT binding assay was performed (**A**) without or (**B**) with paclitaxel on lysate isolated from HEK293T cells transfected with 0N4R WT tau. In the presence of paclitaxel, the same assay was performed on cells expressing tau methylmimetics (**C**) K174F, (**D**) K180F, (**E**) K254F, (**F**) K267F, and (**G**) K369F. Immunoblots were probed with antibodies specific to β-tubulin (clone TUB 2.1) or 3026 antibodies specific to the total tau. S = supernatants; P = pellet fractions. The relative molecular weights are shown on the left. (**H**) The percent of tau bound to MTs was calculated based on semi-quantitative densitometric analysis. One-way ANOVA with Dunnett’s test was performed with N = 3 for each group. *** = *p* < 0.001, ** = *p* < 0.01, * = *p* < 0.05, ns = not statistically significant. Error bars show standard errors of the mean.

HEK293T cells were transfected to express wild-type (WT) 0N4R tau or different tau methylmimetics, and these tau variants were compared based on MT binding properties. Tubulin usually remains soluble after cell lysis, but in the presence of the drug paclitaxel, tubulin is actively stabilized and polymerized into MTs [20,21] that are present in pellet fractions after sedimentation via high-speed centrifugation (Figure 2A,B). This approach can be used to isolate proteins that are associated with MTs, such as tau protein, and compare relative MT binding between different proteins or variants. For WT tau without paclitaxel treatment, tubulin primarily remains in the soluble fraction, and almost none of the tau is in the pellet fraction (Figure 2A). After the addition of paclitaxel, most of the tubulin is shifted in the pellet fraction, and tau that binds to MTs can also be found and isolated in the pellet fraction (Figure 2A,B). 

For tau methylmimetic sites located in the proline-rich domain, K174F tau showed significantly decreased MT binding compared with WT tau, while K180F tau was not statistically different from WT tau (Figure 2C,D,H). For methylmimetics within the MT binding region, K267F tau presented with decreased MT binding relative to WT tau, while K254F tau was not significantly different from WT tau (Figure 2E,F,H). K369F tau, which is just downstream of the MT binding region, also resulted in decreased MT binding (Figure 2G,H). 

### 2.3. Single-Site Tau Methylmimetics within WT Tau Did Not Affect Tau Aggregation

**Figure 3:**
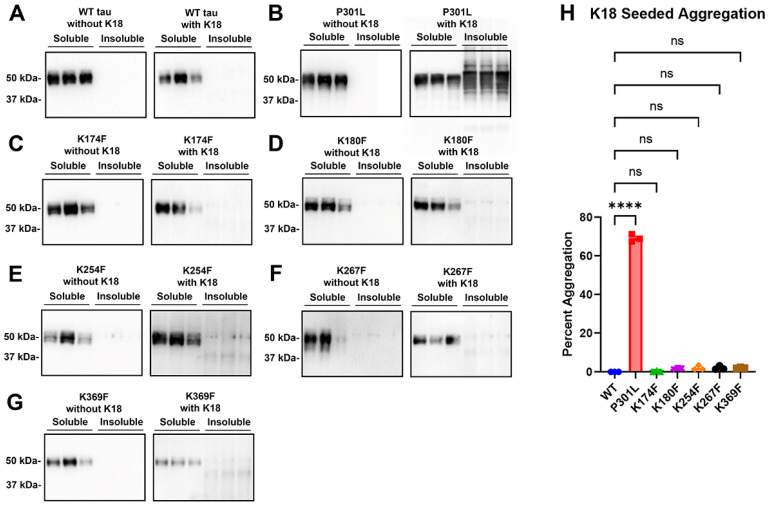
**Single-site tau methylmimetics do not significantly affect intrinsic or prion-type seeded tau aggregation**. HEK293T cells were transfected to express either 0N4R WT tau or tau methylmimetics. A cell-based tau aggregation assay was performed either untreated or with the addition of K18 fibrils on cells transfected to express (**A**) WT, (**B**) P301L, (**C**) K174F, (**D**) K180F, (**E**) K254F, (**F**) K267F, or (**G**) K369F human 0N4R tau. The P301L tau mutant was used as a positive control for seed-induced aggregation. Immunoblots were probed with antibody 3026 for the total tau. The relative molecular weight markers are shown on the left. (**H**) The graph shows the percent aggregation of WT tau, P301L, and different tau methylmimetics. One-way ANOVA with Dunnett’s test was performed with N = 3 for all groups. **** = *p* < 0.0001, ns = not statistically significant. Error bars show standard errors of the mean.

HEK293T cells were transfected to express WT tau or tau methylmimetics and assessed with a cell-based tau aggregation assay. In this assay, cell lysates are fractionated into Triton-soluble and Triton-insoluble fractions to isolate tau aggregates, and the percent of tau aggregation can be calculated based on a ratio of the insoluble fraction to the sum of the soluble and insoluble fractions. Tau methylmimetics were compared to WT tau, which did not display significant accumulation in the insoluble fraction and almost all of the detectable tau was within the soluble fraction (Figure 3A). This was true for WT tau with or without the addition of K18 fibrillar seeds, which consist of the central tau domain with all four major MT binding repeats. Tau mutation P301L was used as a positive control because it can cause familial forms of FTD [2,22] and is susceptible to prion-like aggregation after seed induction [23,24,25]. Indeed, P301L did not aggregate significantly by itself but showed significant aggregation after the addition of K18 seeds (Figure 3B). Tau methylmimetics K174F, K180F, K254F, K267F, and K369F did not significantly aggregate with or without K18 seeds (Figure 3C–H). 

### 2.4. Tau Methylmimetics Enhance Prion-like Seeded Aggregation in the Context of the P301L Tau Mutation

**Figure 4:**
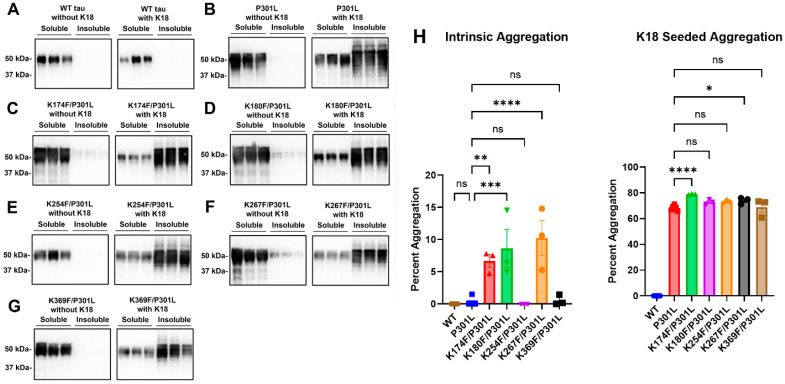
**Tau methylmimetics enhance the prion-like seeded aggregation of tau with the P301L mutation.** Cell-based tau aggregation assays were performed to determine intrinsic and K18-seeded aggregation in cells expressing (**A**) WT 0N4R tau or 0N4R tau mutants (**B**) P301L, (**C**) K174F/P301L, (**D**) K180F/P301L, (**E**) K254F/P301L, (**F**) K267F/P301L, and (**G**) K369F/P301L. Immunoblots were probed with tau antibody 3026. The relative molecular weight markers are on the left. (**H**) The graph displays the percent aggregation of WT tau and different tau mutations. One-way ANOVA with Dunnett’s test was performed with N = 6 for WT tau and P301L and N = 3 for all other mutations. **** = *p* < 0.0001, *** = *p* < 0.001, ** = *p* < 0.01, * = *p* < 0.05, ns = not statistically significant. Error bars show standard errors of the mean.

While tau methylmimetics are not sufficient to induce tau aggregation, they may act as contributing factors and modulators of tau aggregation. To assess the effects of pseudomethylation on pathogenic tau, tau methylmimetics K174F, K180F, K254F, K267F, and K369F were added to the P301L tau mutation in the same plasmid. Compared with P301L tau (Figure 4B), the combination of K174F/P301L, K180F/P301L, and K267F/P301L showed statistically significant increases in intrinsic aggregation (Figure 4C,D,F,H). When cells were treated with exogenous K18 seeds, K174F/P301L tau and K267F/P301L tau also presented with enhanced tau aggregation compared with P301L tau (Figure 4C,F,H). K180F/P301L, K254F/P301L, and K369F/P301L tau were not significantly different from P301L tau in prion-like aggregation after K18 seed induction (Figure 4D,E,G,H).

## 3. Discussion

Tau is an intrinsically disordered protein with a variety of conformations, and these types of proteins are particularly vulnerable to multiple PTMs [26,27]. Different patterns of PTMs represent a variety of proteoforms that can present as differentiating forms of pathogenic tau involved in diverse tauopathies [7,8]. Among these PTMs, tau methylation is less studied, but disease-specific methylation sites on tau filaments isolated from AD patients have been identified [13]. In this study, methylmimetics were used to model four of these major tau methylation sites at important lysines, including K174, K180, K254, and K267, as well as a potential methylation site, K369. 

Other PTMs, including acetylation and phosphorylation, have been known to regulate tau–MT interactions [7,14,28,29]. In this study, we extend the notion that specific tau methylation sites contribute to modulating tau–MT interactions. Of the methylmimetics assessed, we found that three different sites (K174F, K267F, and K369F) decreased tau–MT binding. K174 is within the proline-rich region, which is important for regulating MT functions [30]. Additionally, K267 and K369 are within or close to the MT binding region that directly interacts with the MT interface [31,32]. These findings demonstrate that not all methylation sites within these regions can directly affect MT interactions since K180 in the proline-rich region and K254 in the MT binding region did not alter MT binding. Therefore, it can be surmised that individual methylation sites selectively regulate MT functions. 

Prior in vitro studies involving bulk reductive methylation led to overall decreased tau aggregation in terms of both the initiation and elongation phases [9]. This effect could be due to the excessive methylation of multiple sites, and the methylation of individual sites is better able to model its effects on tau aggregation. Tau methylmimetics by themselves did not significantly alter aggregation propensity. However, in the context of the pro-aggregation tau mutation P301L, tau methylmimetics K174F and K267F showed increased tau aggregation after exogenous treatment with K18 seeds. In addition, K174F, K180F, and K267F also increased intrinsic tau aggregation when combined with P301L compared with P301L tau. Within the timeframe of the current studies, single tau methylmimetics alone are not sufficient to initiate tau aggregation, but they can enhance tau aggregation after the process has started or is primed by an initiator of tau misfolding. 

A limitation of this study is that tau pseudomethylation does not perfectly capture all the properties of true lysine methylation and can only approximate size and charge. However, pseudomethylation is the best available method to study site-specific tau methylation since few lysine methyltransferases have been identified that act on tau, and multiple enzymes might be involved in methylation reactions [10]. In addition, we cannot exclude the possibility that some effects of methylmimetics may be due to the prevention of other PTMs, such as acetylation and ubiquitination, which also compete for the same lysine residues [10,13]. 

Overall, the studies on tau methylmimetics have effectively demonstrated that specific lysine sites are important modulators of both MT binding and tau aggregation. In addition to phosphorylation, acetylation, and other modifications, tau methylation contributes to the unique pattern of PTMs found in different tauopathies and will be a potential drug target for immunotherapy and other tau-targeting therapies.

## 4. Materials and Methods

### 4.1. K18 Protein Purification and Fibrillization

Tau fibrillar seeds were generated from the tau protein fragment K18, which contains the four major MT binding repeats, from Q244 to E372 (numbering is based on 2N4R full-length tau), as described in prior studies [24,33]. K18 tau was expressed in the BL21 (DE3)/RIL *Escherichia coli* bacterial strain (New England Biolabs, Ipswich, MA, USA) using the bacterial plasmid pRK172 with the respective tau cDNA inserts but also including an additional N-terminal ATG codon. After growing bacterial cultures in the presence of the antibiotic ampicillin and the induction of tau expression with isopropyl β-d-1 thiogalactopyranide, the bacteria samples were pelleted via centrifugation and resuspended in high-salt RAB buffer (0.1 M MES, pH 7.0, 1 mM EGTA, 0.5 mM MgSO_4_, 750 mM NaCl, 20 mM NaF) with a cocktail of protease inhibitors (1 mM phenylmethylsulfonyl fluoride and 1 μg/mL each of pepstatin, leupeptin, *N*-tosyl-l-phenylalanyl chloromethyl ketone, *N*-tosyl-lysine chloromethyl ketone, and soybean trypsin inhibitor). After homogenization and heating to 100 °C for 10 min, the bacterial debris was pelleted for 20 min at 14,000× *g* via centrifuge. The supernatant was filtered with a 0.22-micron filtration device and dialyzed overnight in fast protein liquid chromatography (FPLC) buffer A (20 mM PIPES, pH 6.5, 10 mM NaCl, 2 mM DTT, 1 mM EGTA, 1 mM MgSO_4_, 0.1 mM PMSF). Dialyzed samples were concentrated and purified on a Resource S column via FPLC using an NGC chromatography system (Biorad, Hercules, CA, USA) with a NaCl gradient of 0.5 M. The fractions containing tau proteins were confirmed by sodium dodecyl sulfate-polyacrylamide gel electrophoresis (SDS-PAGE) and Coomassie R250 staining. These fractions were then pooled, concentrated, and dialyzed in 20 mM MOPS, pH 6.0. Final protein concentrations were determined with a bicinchoninic acid assay using bovine serum albumin (Thermo Fisher Scientific, Waltham, MA, USA) as the standard, and the output was read using a colorimetric assay. 

To create small tau fibrils, K18 tau protein was added to sterile PBS at 1 mg/mL with 50 μM heparin as an anionic inducer of tau aggregation. The mixture was placed in a shaker at 1050 RPM and 37 °C for over two days. To remove the remaining heparin, K18 fibrils were then centrifuged at 100,000× *g* for 30 min and resuspended in sterile PBS. The purified K18 was sonicated in a water bath for 1 h to create short fibrils [24,33]. 

### 4.2. Plasmid Cloning and Site-Directed Mutagenesis

The 0N4R human tau cDNA isoform was cloned into plasmid vector pcDNA3.1(+). Tau methylation mimetics K174F, K180F, K254F, K267F, and K369F were created by mutating the codons for lysines into phenylalanines. Combined constructs K174F/P301L, K180F/P301L, K254F/P301L, K267F/P301L, and K369F/P301L were generated by adding the P301L tau mutation to each methylmimetic in the same plasmid. All of the plasmid constructs were created via site-directed mutagenesis using primers with partially overlapping oligonucleotides during a polymerase chain reaction (PCR) [34]. Q5 high-fidelity DNA polymerase (New England Biolabs, Ipswich, MA, USA) was used for site-specific mutagenesis, and PCR reaction mixes contained DNase-free water, Q5 reaction buffer, dNTP nucleotides, forward and reverse primers, and a plasmid template. The restriction enzyme DpnI (New England Biolabs, Ipswich, MA, USA) was used to digest any residual parental dsDNA. The resulting DNA constructs with tau mutants were identified and confirmed via DNA sequencing as a service by Genewiz (South Plainfield, NJ, USA). 

### 4.3. Cell Culture and Calcium Phosphate Transfection

HEK293T cells were maintained and grown in Dulbecco’s modified eagle media with 10% fetal bovine serum at 37 °C and 5% CO_2_ with added antibiotics (100 U/mL penicillin, 100 mg/mL streptomycin). Cell transfection was performed by using calcium phosphate precipitation, as previously described [24,35]. Cells were split equally into 12-well plates at ~20% confluency. For each well, 1.5 µg of DNA was mixed with 18.75 µL of 0.25 M CaCl_2_. This mixture was added to an equal amount of 2X BES buffer (50 mM BES, 280 mM NaCl, 1.5 mM Na_2_HPO_4_, pH 6.96) and incubated for 15 to 20 min at room temperature. The final solution was added dropwise to each well of cells.

For the cell-seeding studies to study the tau aggregation, 1 µM of K18 tau fibrils (based on the molecular weight of K18 tau peptide) was added to transfected cells one hour after transfection. At 16 h after transfection, cells were washed with PBS, and fresh DMEM in 3% FBS was added to each well. Cells were harvested 48 h after the media change and processed for immunoblotting. 

### 4.4. Cell-Based MT Binding Assay

HEK293T cells were lysed in 200 μL of PEM buffer (80 mM PIPES, pH 6.8, 1 mM EGTA, 1 mM MgCl_2_) supplemented with 0.1% Triton X-100 detergent, 2 mM GTP, 20 μM of paclitaxel, and a mix of protease inhibitors (1 mM phenylmethylsulfonyl fluoride and 1 μg/mL each of pepstatin, leupeptin, *N*-tosyl-l-phenylalanyl chloromethyl ketone, *N*-tosyl-lysine chloromethyl ketone, and soybean trypsin inhibitor) [24,33,36]. Cell lysates were incubated in a 37 °C water bath for 30 min and then sedimented at 100,000× *g* for 30 min to isolate MTs in the pellet. The supernatant was transferred to a new tube and the pellet (MT fraction with bound proteins) was resuspended in PEM buffer. The pellet fraction was homogenized and SDS sample loading buffer (10 mM Tris, pH 6.8, 1 mM EDTA, 40 mM DTT, 0.005% bromophenol blue, 0.0025% pyronin yellow, 1% SDS, 10% sucrose) was added to both fractions. Equal amounts of supernatant and pellet were loaded in each lane on acrylamide gels for immunoblotting. The percent of MT-bound tau was calculated as tau in the pellet fraction/(supernatant fraction + pellet fraction) × 100. 

### 4.5. Cell-Based Tau Aggregation Assay

HEK293T cells were processed for cellular lysis in 200 μL of Triton Lysis Buffer (25 mM Tris-HCl, pH 7.5, 150 mM NaCl, 1mM EDTA, 1% Triton X-100, 20 mM NaF) with a mix of protease inhibitors (1 mM phenylmethylsulfonyl fluoride and 1 μg/mL each of pepstatin, leupeptin, *N*-tosyl-l-phenylalanyl chloromethyl ketone, *N*-tosyl-lysine chloromethyl ketone, and soybean trypsin inhibitor) [24,33]. Cell lysates were sedimented at 100,000× *g* and 4 °C for 30 min. The supernatants were collected as Triton-soluble fractions. The pellets or the Triton-insoluble fractions were washed with additional buffer and centrifuged again at 100,000× *g* and 4 °C for 30 min. After the wash step, the pellets were reconstituted in Triton Lysis Buffer. SDS loading buffer (final concentration of 10 mM Tris, pH 6.8, 1 mM EDTA, 40 mM DTT, 0.005% bromophenol blue, 0.0025% pyronin yellow, 1% SDS, 10% sucrose) was added to both the Triton-soluble and -insoluble fractions, which were heated at 95 °C for 10 min. The Triton-insoluble fraction was probe-sonicated and heated at 95 °C again for 10 min. Percent aggregation was calculated as a function of tau in the insoluble fraction/(insoluble fraction + soluble fraction) × 100.

### 4.6. Immunoblotting

Subsequently, 10% polyacrylamide gels were prepared using Biorad gel apparatuses and a mix of reagents, including acrylamide, 1.5 M Tris pH 8.8, 10% sodium dodecyl sulfate (SDS), and 10% ammonium persulfate (APS) and polymerized by the catalyst tetramethylethylenediamine (TEMED). A separate stacking gel was made using 0.5 M Tris pH 6.8, acrylamide, APS, SDS, and TEMED. Equal amounts of each sample were loaded on 10% polyacrylamide gels and separated using SDS-PAGE. After electrophoretic transfer, the membranes were blocked in 5% milk with Tris-buffered saline (TBS) solution (50 mM Tris, 150 mM NaCl, pH 7.5) for an hour and incubated in primary antibody overnight at 4 °C. Clone TUB 2.1 is a mouse monoclonal antibody against β-tubulin (Sigma-Aldrich, St. Louis, MO, USA), and 3026 tau antibody is a rabbit polyclonal antibody that detects total human tau [37]. After TBS washes, goat anti-rabbit or anti-mouse secondary antibodies conjugated to horseradish peroxidase (Jackson Immuno Research labs, Westgrove, PA, USA) were added to the membranes for an hour. After several TBS washes, the membranes were exposed and imaged using Western Lightning Plus ECL reagents (PerkinElmer, Waltham, MA, USA). Each lane was semi-quantitatively measured in the ImageJ 2 software using densitometric analysis [38]. Statistical tests were calculated using the GraphPad Prism program for one-way analysis of variance (ANOVA) with post hoc analysis and Dunnett’s test. 

## Data Availability

Data is available within the article.

## References

[1] (2022). 2022 Alzheimer’s Disease Facts and Figures. Alzheimers Dement..

[2] Wang Y., Mandelkow E. (2016). Tau in Physiology and Pathology. Nat. Rev. Neurosci..

[3] Kadavath H., Hofele R.V., Biernat J., Kumar S., Tepper K., Urlaub H., Mandelkow E., Zweckstetter M. (2015). Tau Stabilizes Microtubules by Binding at the Interface between Tubulin Heterodimers. Proc. Natl. Acad. Sci. USA.

[4] Weingarten M.D., Lockwood A.H., Hwo S.Y., Kirschner M.W. (1975). A Protein Factor Essential for Microtubule Assembly. Proc. Natl. Acad. Sci. USA.

[5] Iqbal K., Liu F., Gong C.-X. (2016). Tau and Neurodegenerative Disease: The Story so Far. Nat. Rev. Neurol..

[6] Zhang Y., Wu K.-M., Yang L., Dong Q., Yu J.-T. (2022). Tauopathies: New Perspectives and Challenges. Mol. Neurodegener..

[7] Alquezar C., Arya S., Kao A.W. (2020). Tau Post-Translational Modifications: Dynamic Transformers of Tau Function, Degradation, and Aggregation. Front. Neurol..

[8] Wesseling H., Mair W., Kumar M., Schlaffner C.N., Tang S., Beerepoot P., Fatou B., Guise A.J., Cheng L., Takeda S. (2020). Tau PTM Profiles Identify Patient Heterogeneity and Stages of Alzheimer’s Disease. Cell.

[9] Funk K.E., Thomas S.N., Schafer K.N., Cooper G.L., Liao Z., Clark D.J., Yang A.J., Kuret J. (2014). Lysine Methylation Is an Endogenous Post-Translational Modification of Tau Protein in Human Brain and a Modulator of Aggregation Propensity. Biochem. J..

[10] Balmik A.A., Chinnathambi S. (2021). Methylation as a Key Regulator of Tau Aggregation and Neuronal Health in Alzheimer’s Disease. Cell Commun. Signal.

[11] Bichmann M., Prat Oriol N., Ercan-Herbst E., Schöndorf D.C., Gomez Ramos B., Schwärzler V., Neu M., Schlüter A., Wang X., Jin L. (2021). SETD7-Mediated Monomethylation Is Enriched on Soluble Tau in Alzheimer’s Disease. Mol. Neurodegener..

[12] Huseby C.J., Hoffman C.N., Cooper G.L., Cocuron J.C., Alonso A.P., Thomas S.N., Yang A.J., Kuret J. (2019). Quantification of Tau Protein Lysine Methylation in Aging and Alzheimer’s Disease. J. Alzheimers Dis..

[13] Thomas S.N., Funk K.E., Wan Y., Liao Z., Davies P., Kuret J., Yang A.J. (2012). Dual Modification of Alzheimer’s Disease PHF-Tau Protein by Lysine Methylation and Ubiquitylation: A Mass Spectrometry Approach. Acta Neuropathol..

[14] Xia Y., Prokop S., Giasson B.I. (2021). “Don’t Phos Over Tau”: Recent Developments in Clinical Biomarkers and Therapies Targeting Tau Phosphorylation in Alzheimer’s Disease and Other Tauopathies. Mol. Neurodegener..

[15] Kontaxi C., Piccardo P., Gill A.C. (2017). Lysine-Directed Post-Translational Modifications of Tau Protein in Alzheimer’s Disease and Related Tauopathies. Front. Mol. Biosci..

[16] Huq M.D.M., Tsai N.-P., Khan S.A., Wei L.-N. (2007). Lysine Trimethylation of Retinoic Acid Receptor-α. Mol. Cell. Proteom..

[17] Chung H.H., Sze S.K., En Woo A.R., Sun Y., Sim K.H., Dong X.M., Lin V.C.L. (2014). Lysine Methylation of Progesterone Receptor at Activation Function 1 Regulates Both Ligand-Independent Activity and Ligand Sensitivity of the Receptor. J. Biol. Chem..

[18] Huq M.D.M., Ha S.G., Wei L.N. (2008). Modulation of Retinoic Acid Receptor Alpha Activity by Lysine Methylation in the DNA Binding Domain. J. Proteome Res..

[19] Neumann M., Schulz-Schaeffer W., Anthony Crowther R., Smith M.J., Spillantini M.G., Goedert M., Kretzschmar H.A. (2001). Pick’s Disease Associated with the Novel Tau Gene Mutation K369I. Ann. Neurol..

[20] Vallee R.B. (1982). A Taxol-Dependent Procedure for the Isolation of Microtubules and Microtubule-Associated Proteins (MAPs). J. Cell Biol..

[21] Kumar N. (1981). Taxol-Induced Polymerization of Purified Tubulin. Mechanism of Action. J. Biol. Chem..

[22] Hutton M., Lendon C.L., Rizzu P., Baker M., Froelich S., Houlden H., Pickering-Brown S., Chakraverty S., Isaacs A., Grover A. (1998). Association of Missense and 5’-Splice-Site Mutations in Tau with the Inherited Dementia FTDP-17. Nature.

[23] Combs B., Gamblin T.C. (2012). FTDP-17 Tau Mutations Induce Distinct Effects on Aggregation and Microtubule Interactions. Biochemistry.

[24] Xia Y., Sorrentino Z.A., Kim J.D., Strang K.H., Riffe C.J., Giasson B.I. (2019). Impaired Tau-Microtubule Interactions Are Prevalent among Pathogenic Tau Variants Arising from Missense Mutations. J. Biol. Chem..

[25] Strang K.H., Croft C.L., Sorrentino Z.A., Chakrabarty P., Golde T.E., Giasson B.I. (2018). Distinct Differences in Prion-like Seeding and Aggregation between Tau Protein Variants Provide Mechanistic Insights into Tauopathies. J. Biol. Chem..

[26] Skrabana R., Sevcik J., Novak M. (2006). Intrinsically Disordered Proteins in the Neurodegenerative Processes: Formation of Tau Protein Paired Helical Filaments and Their Analysis. Cell. Mol. Neurobiol..

[27] Bah A., Forman-Kay J.D. (2016). Modulation of Intrinsically Disordered Protein Function by Post-Translational Modifications. J. Biol. Chem..

[28] Gorsky M.K., Burnouf S., Sofola-Adesakin O., Dols J., Augustin H., Weigelt C.M., Grönke S., Partridge L. (2017). Pseudo-Acetylation of Multiple Sites on Human Tau Proteins Alters Tau Phosphorylation and Microtubule Binding, and Ameliorates Amyloid Beta Toxicity. Sci. Rep..

[29] Xia Y., Bell B.M., Giasson B.I. (2021). Tau K321/K353 Pseudoacetylation within KXGS Motifs Regulates Tau–Microtubule Interactions and Inhibits Aggregation. Sci. Rep..

[30] Goode B.L., Denis P.E., Panda D., Radeke M.J., Miller H.P., Wilson L., Feinstein S.C. (1997). Functional Interactions between the Proline-Rich and Repeat Regions of Tau Enhance Microtubule Binding and Assembly. Mol. Biol. Cell.

[31] Lee G., Neve R.L., Kosik K.S. (1989). The Microtubule Binding Domain of Tau Protein. Neuron.

[32] Kellogg E.H., Hejab N.M.A., Poepsel S., Downing K.H., DiMaio F., Nogales E. (2018). Near-Atomic Model of Microtubule-Tau Interactions. Science.

[33] Xia Y., Nasif L., Giasson B.I. (2021). Pathogenic MAPT Mutations Q336H and Q336R Have Isoform-dependent Differences in Aggregation Propensity and Microtubule Dysfunction. J. Neurochem..

[34] Zheng L. (2004). An Efficient One-Step Site-Directed and Site-Saturation Mutagenesis Protocol. Nucleic Acids Res..

[35] Xia Y., Prokop S., Gorion K.-M.M., Kim J.D., Sorrentino Z.A., Bell B.M., Manaois A.N., Chakrabarty P., Davies P., Giasson B.I. (2020). Tau Ser208 Phosphorylation Promotes Aggregation and Reveals Neuropathologic Diversity in Alzheimer’s Disease and Other Tauopathies. Acta Neuropathol. Commun..

[36] Vogelsberg-Ragaglia V., Bruce J., Richter-Landsberg C., Zhang B., Hong M., Trojanowski J.Q., Lee V.M.-Y. (2000). Distinct FTDP-17 Missense Mutations in Tau Produce Tau Aggregates and Other Pathological Phenotypes in Transfected CHO Cells. Mol. Biol. Cell.

[37] Strang K.H., Goodwin M.S., Riffe C., Moore B.D., Chakrabarty P., Levites Y., Golde T.E., Giasson B.I. (2017). Generation and Characterization of New Monoclonal Antibodies Targeting the PHF1 and AT8 Epitopes on Human Tau. Acta Neuropathol. Commun..

[38] Schneider C.A., Rasband W.S., Eliceiri K.W. (2012). NIH Image to ImageJ: 25 Years of Image Analysis. Nat. Methods.

